# The Influence of Anticipated HIV Stigma on Health-related Behaviors, Self-rated Health, and Treatment Preferences Among People Living with HIV in East Asia

**DOI:** 10.1007/s10461-022-03865-5

**Published:** 2022-11-08

**Authors:** Fei Yu, Yu-Hsiang Hsiao, Sejun Park, Keita Kambara, Brent Allan, Garry Brough, Ta-Fen Hwang, Nathalie Dang, Benjamin Young, Rickesh Patel, Andres Maldonado, Chinyere Okoli

**Affiliations:** 1 Danlan Beijing Media Limited, Beijing, China; 2 Positive Alliance, Taipei, Taiwan; 3 Love4One, Seoul, South Korea; 4 Japanese Network of People living with HIV/AIDS, Tokyo, Japan; 5 Australasian Society for HIV, Viral Hepatitis and Sexual Health Medicine, Sydney, Australia; 6 International Council of AIDS Service Organizations (ICASO), Toronto, Canada; 7 Positively UK, London, UK; 8 ViiV Healthcare, Singapore, Singapore; 9 ViiV Healthcare, North Carolina, USA; 10 ViiV Healthcare, Brentford, UK; 11 ViiV Healthcare, Wavre, Belgium

**Keywords:** Stigma, Asia, Quality of life, Antiretrovirals, Medication, HIV

## Abstract

Long-acting injectable regimens for HIV treatment have been developed which are less frequent, more discreet, and more desirable for some people living with HIV (PLHIV) and may help reduce stigma-related barriers to HIV treatment. However, there is little information on the relationship between reported stigma and preference for these newer treatments. We characterized anticipated, experienced, and internalized HIV stigma and examined the associations with treatment preferences among an international sample of PLHIV. Data came from the international, web-based, cross-sectional study called “Positive Perspectives” conducted among PLHIV aged ≥ 18 years in 25 geographic locations during 2019 (n = 2389). Descriptive analyses were stratified among East Asian (n = 230) vs. non-Asian (n = 2159) participants. Results showed that prevalence of anticipated stigma was significantly higher among East Asian than non-Asian participants (72.2%[166/230] vs. 63.8%[1377/2159], p = 0.011). A significantly higher percentage of East Asian (68.7%[158/230]) than non-Asian participants (43.3%[935/2159] indicated that someone finding their HIV pills would cause them much “stress or anxiety” (p < 0.001). Actions taken by some PLHIV to prevent unwanted disclosure included restricting who they shared their HIV status with, hiding their HIV pills, or even skipping a dose altogether because of privacy concerns. Overall, 50.0%[115/230] East Asian participants believed HIV would reduce their lifespan and 43.0%[99/230] no longer planned for their old age because of HIV. Anticipated stigma was strongly associated with receptivity to non-daily regimens. Concerted efforts to reduce stigma and deliver flexible treatment options that address the unmet treatment needs of PLHIV, including confidentiality concerns, may improve their health-related quality of life.

## Introduction

HIV stigma is a public health concern that can subvert the progress of countries in meeting the UNAIDS 95-95-95 targets of increasing the percentage of people living with HIV (PLHIV) who are diagnosed, on antiretroviral therapy (ART), and virally suppressed to ≥ 95% for each indicator. [1,2] Stigma can undermine the first objective of increasing diagnoses by discouraging individuals at risk from getting tested because they are worried of what people might think of them if their result is positive, or because they do not believe themselves to be the ‘kind of person’ who would get HIV. [3–5] Stigma, especially within healthcare settings, can further undermine the second and third objectives (increasing ART coverage and viral suppression rates respectively) by limiting the uptake and utilization of healthcare services. [6–9] Stigma is also a threat to the proposed fourth objective of achieving good quality of life among PLHIV because of the climate of secrecy, fear, shame, and victimization that it creates.[5,10-12] For example, within the Chinese context, Yang and Kleinman characterized how HIV stigma erodes one’s “moral face” – a “group’s evaluation of a person’s moral reputation, record for fulfilling social exchange obligations, and status as a good human being”. [13].

Relationally, stigma can be described both from the perspective of the person being stigmatized (internalized, anticipated, or experienced stigma), or the person from which the stigma is emanating (stereotypes, prejudice, and discrimination). [14,15] Internalized stigma refers to endorsement and self-acceptance of negative HIV-related assumptions among PLHIV on account of their HIV status. [14] Experienced stigma describes actual exposure to discrimination, devaluation, and prejudice by PLHIV because of their HIV status. [14] Anticipated stigma on the other hand describes the expectation on the part of PLHIV, that others will treat them negatively in future situations on account of their HIV status. [14] Superimposed on HIV stigma is discrimination and stigma towards homosexuality, a significant concern as gay, bisexual and other men who have sex with men are a key population across the East Asia region. [16].

Stigma in healthcare settings could be an unintended consequence of structural or systemic factors (e.g., how the health system is set up). For example, In South Korea, a drug-drug interaction screening program called Drug Utilization Review (DUR) was established [17,18] which registers information on all prescribed drugs as well as analyzes and flags potential drug-drug interactions. While the DUR program has obvious benefits in helping to screen for inappropriate drug use, it also creates the potential for inadvertent disclosure of HIV status because if someone on ART visits a local clinic to receive a prescription for a non-HIV medication, the healthcare provider seeing them could potentially access their current ART within the DUR system and infer their HIV status even if they did not share that information. Such unwanted nondisclosure may trigger apprehension and anticipated stigma among PLHIV, especially when visiting smaller facilities that do not typically provide HIV care. For this reason, some PLHIV may prefer visiting secondary or tertiary hospitals that routinely provide care to PLHIV. National ART prescribing policies may also inadvertently contribute to HIV stigma especially when they deviate from evidence-based global recommendations. [19] For example, despite the WHO’s “treat all” guidelines which recommend that ART be initiated ideally on the very same day HIV is diagnosed whenever possible, [20] some countries in the Asian region still base ART initiation not on diagnosis, but on CD4 count strata. [19] Such clinical practices create the potential where PLHIV might enter care with advanced HIV infection and may never see the benefit of treatment such as achieving undetectable viral loads, which in itself may reinforce internalized stigma. [21,22] According to the 2020 Global AIDS report, only two Asian countries (Cambodia and Thailand) met the 90-90-90 targets by the end of 2019. [23] The wide dispersion in viral suppression rates in the Asian region may be attributable to varied policies around ART prescribing across the region which mean that progress towards viral suppression may be dependent on local policy, rather than evidence-based global recommendations.

Understanding HIV stigma in the Asian region and how it compares to the rest of the world is important as this region has more PLHIV than any other region outside sub-Saharan Africa. [1,24] Within the Asian region, ART coverage is widely variable, from as low as 26% in Indonesia, to 92.9% in China, [25,26] underscoring the need to examine contextual cross-country differences in the region, in addition to any comparisons between the region and the rest of the world. In 2011, UNAIDS published a report on the HIV stigma index in in nine countries in Asia and the Pacific (Bangladesh, Cambodia, China, Fiji, Myanmar, Pakistan, Philippines, Sri Lanka, Thailand). [27] This report provided the first large-scale regional comparison of standardized HIV-related stigma indicators. However, except for China, no East Asian country was included in this UNAIDS report. Given the impressive diversity of the East Asian region, a better understanding of HIV stigma within the region, and how it compares to the rest of the world, is key to guiding public health policy, programs aimed at curbing stigma. Furthermore, much of the research on stigma in the Asian region has examined the topic from an ethnographic, social, societal, or interpersonal perspective. [9,13,28] Missing from this narrative is the potential role of new HIV treatments to alleviate stigma among PLHIV. The HIV landscape has changed with the emergence of new treatment modalities such as longer-acting treatments that no longer require daily oral dosing. [29] Long-acting ART represents an important shift in treatment options and could play a significant role in reducing stigma among PLHIV. In a recent international survey of PLHIV, 87.6% of those with confidentiality concerns regarding their HIV status believed long-acting ART would help mitigate such confidentiality concerns. [30] Understanding the associations between anticipated stigma and preferences for these newer regimens can help healthcare providers develop treatment plans that take into account the full spectrum of PLHIV’s concerns, values, and preferences in line with person-centered care. [31[ To better characterize HIV stigma multi-contextually and to examine the associations with treatment preferences, this study used data from an international survey of PLHIV on ART in 25 geographic locations to: (1) analyze indicators of anticipated, internalized and experienced stigma and compare between surveyed PLHIV in the East Asian region vs. the rest of the world; (2) explore the relationship between reported stigma among PLHIV and preference for new HIV treatments.

## Methods

### Data Source

Data came from the web-based, cross-sectional study called “Positive Perspectives” that was conducted among PLHIV on ART aged ≥ 18 years in 25 geographic locations during 2019 (n = 2389).[32-36] PLHIV were sampled non-probabilistically from various sources, including existing panels of confirmed HIV sero-positive individuals, HIV charities or support groups, online HIV communities, and social media platforms. Of the 2389 participants, n = 230 came from the East Asian region (China, Japan, South Korea and Taiwan) while n = 2159 were outside the Asian region (Argentina, Australia, Austria, Belgium, Brazil, Canada, Chile, France, Germany, Ireland, Italy, Mexico, Netherlands, Poland, Portugal, Russia, South Africa, Spain, Switzerland, Switzerland, United Kingdom, and the United States).

### Measures

#### Stigma Indicators

**Fig. 1:**
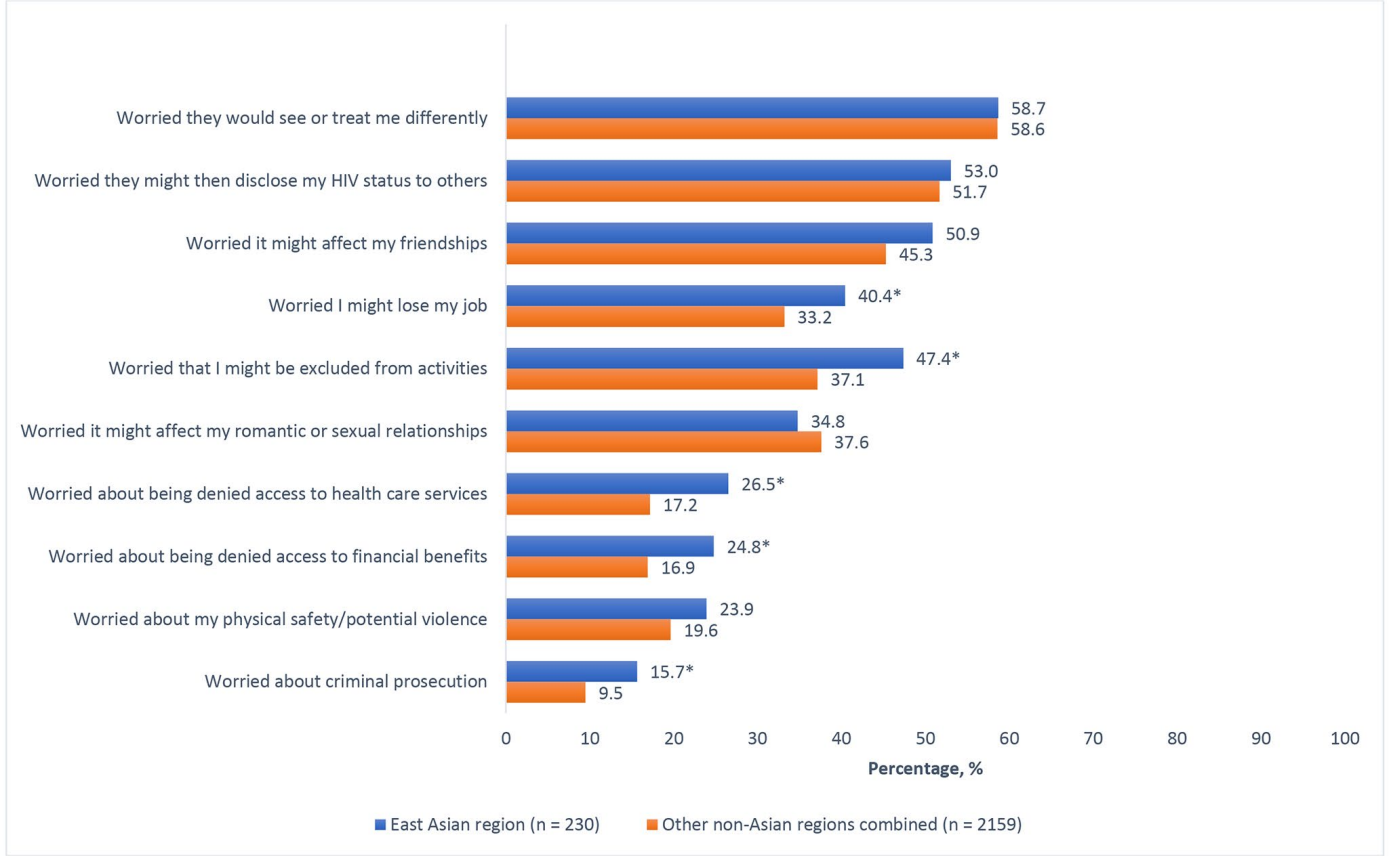
Percentage of people living with HIV who reported various reasons for not sharing their HIV status in the past, stratified by East Asian (N = 230) or non-Asian region (N = 2159). (* P < 0.05)

We explored surrogate markers for anticipated and internalized stigma. Participants were classified as having anticipated stigma if they reported they were uncomfortable “sharing [their] HIV status” and cited at least one discrimination-related reason for past nondisclosure (reasons shown in Fig. 1). To capture anticipated stigma within healthcare settings specifically, we analyzed the indicator for whether participants had ever withheld their HIV status from someone because they were afraid of “being denied access to health care services”. To assess the potential role of aspects of treatment in triggering anticipated stigma, participants were asked how anxious or stressed they would feel if someone saw their HIV pills, whether they had ever hidden or disguised their HIV pills in the past 6 months to avoid revealing their HIV status, and whether they had shared their HIV status within different settings, including with physicians, nurses and other healthcare personnel other than their main HIV care provider.

Data were also collected on surrogate markers for internalized stigma. Two such indicators measuring poor self-prognosis for HIV mortality were the statements: “HIV will reduce my life span” and “Because of my HIV, I do not plan for my old age”. An affirmative response (“Agree” or “Strongly agree”) to at least one of these two indicators was taken as a manifestation of internalized stigma because it suggested that despite evidence showing comparable life expectancy between the general population and PLHIV on ART, [37] such individuals had endorsed and self-affirmed the stigma-linked narrative that they would die prematurely from HIV. We also explored how daily ART dosing impacted negative sentiments of oneself using the following self-reported measures: “Taking pills for HIV every day is a daily reminder of HIV in my life” and “Taking pills for HIV every day is a link to some bad memories from my past”.

#### Viral load, Openness to new HIV Regimens and Communication with Providers

Self-reported virologic control was defined as a response of “undetectable” or “suppressed” versus “detectable,” “unsuppressed,” “I don’t know,” or “prefer not to say” to the question, “What is your most recent viral load?” The survey also collected data on different reasons for missing ART within the past 30 days. Willingness to switch to a longer-acting (non-daily) regimen was defined as a response of “Agree” or “Strongly agree” to the statement “As long as my HIV stays suppressed, I would prefer not having to take HIV medicine every day”. Willingness to switch to a regimen with fewer medicines was an affirmative response to the statement, “As long as my viral load is suppressed, I am open to taking an HIV treatment composed of fewer medicines”. Patient-provider communication was measured by self-reported extent of information sharing and discussion about salient treatment issues (e.g., if their healthcare provider had ever told them about “Undetectable Equals Untransmittable” or “U = U”).

#### Gender and Sexual Orientation Status

Designation of participants as men who have sex with men (MSM) or men who have sex with women (MSW) was derived from two separate variables for self-classified gender and sexual orientation. Individuals who identified their gender as ‘Man (including transman)’, and their sexual orientation as ‘Homosexual/Gay/Lesbian’ were classified as MSM. Individuals who identified their gender as ‘Man (including transman)’ and their sexual orientation as ‘Heterosexual/Straight’ were classified as MSW. All women, regardless of sexual orientation, were grouped into one category because of small sample sizes. Those with unidentified gender or sexual orientation were classified as ‘Undisclosed or unknown’.

### Analyses

Prevalence estimates were computed and compared using chi-square tests. The variability in prevalence estimates across regions was measured with the coefficient of variation. Building on a previous study that showed PLHIV participants from East Asia reported the lowest treatment satisfaction compared to every other region assessed and were also the least likely to share their HIV status with health professionals providing non-HIV care, [35] we explored prevalence and correlates of anticipated stigma within healthcare settings among East Asian participants.

Within the pooled sample, multivariable logistic regression analysis was used to explore factors associated with the different reported reasons for withholding HIV status (surrogate indicators for anticipated stigma). The independent variables were age, gender/sexual orientation; geographic region, year of HIV diagnosis, domicile, education, self-reported viral load and U = U awareness status. The latter was included on the premise that ignorance and fear are the key drivers of stigma, i.e., ignorance about how HIV is transmitted fuels fear of PLHIV (or even by PLHIV) that they might transmit disease, and hence, counseling about U = U could be empowering in mitigating anticipated stigma by PLHIV. [38–40].

We hypothesized that the number of stigma stressors would be associated with preference for regimens that reduced privacy concerns or the risk of disclosure. As a proxy for the number of stigma stressors, we tallied the different contexts in which participants refused to share their HIV status to avoid discrimination (range: 0 to 10, Fig. 1). We regressed openness to using non-daily regimens as a function of the number of stigma stressors within the pooled sample, adjusting for geographic region, duration of HIV, and gender. To determine whether any observed receptivity was specific to that regimen type and not just to new regimens in general, we performed sensitivity analyses with a different outcome — openness to ART with fewer medicines — another novel HIV treatment with less HIV medicines but still administered daily, adjusting for the same variables as before. We also explored whether the odds of reporting different reasons for missing ART within the past 30 days varied by anticipated stigma (binary indicator) after adjusting for geographic region, duration of HIV, and gender. Because of sample size considerations, all multivariable analyses were performed on the full dataset of 2389 participants from all 25 study sites. Statistical analyses were performed in Stata V 14 at p < 0.05.

## Results

### Characteristics of the Study Population

Mean age for East Asian vs. non-Asian participants was 38.7 (SD = 11.3) years vs. 41.5 (SD = 12.2) years respectively; mean HIV duration was 6.4 (SD = 6.2) years vs. 10.5 (SD = 9.8) years respectively (all p < 0.01). Overall, East Asian participants reported lower viral suppression (66.1%[152/230]) than non-Asian participants (74.9%[1618/2159], p = 0.004). Prevalence of self-reported viral suppression also varied more in the East Asian region (coefficient of variation = 33.8%) than the non-Asian region (coefficient of variation = 19.7%). Other characteristics are shown in Table 1.

**Table 1:** Percentage of participants who reported various indicators of stigma and other emotional and psychosocial challenges with HIV medicine among people living with HIV, stratified by Asian (N = 230) and non-East Asian (N = 2159) regions, 2019

Characteristic	Category	N	Anticipated Stigma	Would be anxious or stressed if someone saw their HIV medicine ^a^	Ever disguised or hid their HIV medicine in past 6 months ^b^	Stressed by their daily ART dosing schedule	Daily HIV medicines remind them daily of HIV	Daily ART dosing linked to bad memories	Perceive Daily dosing limits life	Have difficulty swallowing pills	Open to ART with less medicines	Open to non- daily ART
	EAST ASIA	n	%	%	%	%	%	%	%	%	%	%
	Total	230	72.2	68.7	77.0	49.1	53.9	42.2	44.3	43.5	65.2	57.8
Age, years	< 50	180	72.8	69.4	80.0	52.8	58.3	41.1	45.6	43.3	61.1	56.7
	50+	50	70.0	66.0	66.0	36.0	38.0	46.0	40.0	44.0	80.0	62.0
Gender	Male	156	71.8	69.2	73.7	45.5	53.2	34.6	41.0	38.5	69.9	57.1
	Female	73	74.0	67.1	83.6	56.2	54.8	57.5	50.7	54.8	54.8	58.9
	Other	¶	¶	¶	¶	¶	¶	¶	¶	¶	¶	¶
Education	High school or less	35	80.0	68.6	74.3	51.4	42.9	45.7	48.6	31.4	65.7	60.0
	> High school	193	71.0	68.9	77.2	48.7	56.0	42.0	43.0	45.6	65.8	57.5
	Prefer not to answer	¶	¶	¶	¶	¶	¶	¶	¶	¶	¶	¶
Domicile	Metropolitan	134	64.9	68.7	71.6	53.0	54.5	42.5	47.0	41.0	67.2	64.2
	Non-metropolitan	96	82.3	68.8	84.4	43.8	53.1	41.7	40.6	46.9	62.5	49.0
HIV diagnosis year	2017 to 2019	63	74.6	82.5	84.1	60.3	58.7	54.0	55.6	50.8	69.8	66.7
	2010 to 2016	119	76.5	68.9	82.4	48.7	58.8	42.0	48.7	43.7	69.7	54.6
	Pre-2010	48	58.3	50.0	54.2	35.4	35.4	27.1	18.8	33.3	47.9	54.2
	NON-EAST ASIA											
	Total	2159	63.8	43.3	55.9	31.6	58.8	34.4	27.3	32.0	73.0	54.3
Age, years	< 50	1510	66.9	48.9	63.6	36.6	60.9	37.3	31.3	36.4	70.7	54.0
	50+	649	56.5	30.4	37.8	19.9	54.1	27.6	18.0	21.7	78.4	55.2
Gender	Male	1467	64.7	42.2	55.9	29.2	57.0	32.0	25.7	30.8	73.5	54.2
	Female	623	60.7	44.9	55.9	36.4	61.5	38.2	29.4	34.3	71.9	55.5
	Other	69	72.5	52.2	55.1	39.1	73.9	49.3	42.0	34.8	72.5	46.4
Education	High school or less	497	61.8	40.2	53.1	28.8	58.1	33.6	23.9	30.8	71.0	51.1
	> High school	1563	62.8	44.2	56.2	32.2	58.7	34.7	28.0	31.1	75.9	56.6
	Prefer not to answer	99	88.9	44.4	64.6	36.4	63.6	32.3	32.3	51.5	37.4	34.3
Domicile	Metropolitan	1201	65.3	42.4	53.0	31.5	60.4	32.9	24.4	25.6	76.9	56.4
	Non-metropolitan	958	61.9	44.5	59.4	31.7	56.8	36.2	30.9	39.9	68.1	51.8
HIV diagnosis year	2017 to 2019	485	64.7	47.8	75.1	40.4	68.9	43.9	35.3	39.6	73.6	60.8
	2010 to 2016	794	67.6	51.4	63.5	36.3	59.6	35.5	31.1	34.3	68.9	52.4
	Pre-2010	880	59.8	33.5	38.4	22.5	52.6	28.1	19.4	25.7	76.4	52.5

Note: ART = antiretroviral therapy

¶ Estimates not shown because of small sample size.

^a^ Participants were asked, “If someone you did not want to see your HIV pills were to find them, how much stress or anxiety would that cause you, if any?” Scores of ≥ 4 on an ordinal scale from 1–5 were taken as a positive indication of stress/anxiety over inadvertent disclosure.

^b^ Hiding/disguising of HIV medicines was defined as a response of "Yes" to the question "In the past six months, have you ever hidden or disguised your HIV medicine to avoid revealing your status?”

### Indicators of Stigma Among PLHIV

**Fig. 2:**
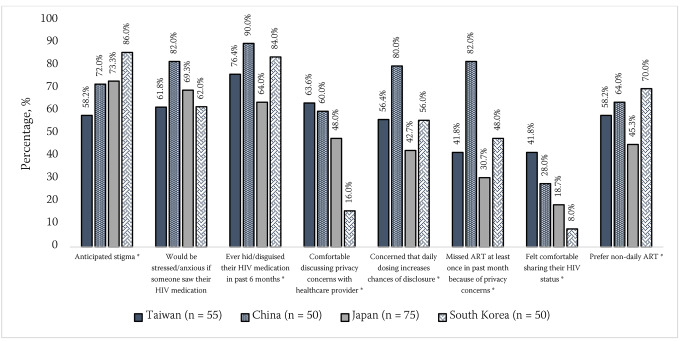
Percentage of participants in the East Asian region who reported anticipated stigma ^a^ as well as other perceptions regarding privacy and confidentiality of HIV identity. (Note: ART = antiretroviral therapy. Asterisks (*) indicate results for which significant overall differences existed by geographic location) ^a^ Participants were classified as reporting anticipated stigma if they reported they were not comfortable with “sharing [their] HIV status” *and* selected at least one of the following sources of worry/concern as a reason for not sharing their HIV status in the past: “criminal prosecution”, “being denied access to financial benefits/support”, “my physical safety/potential violence”, “being denied access to health care services”, “it might affect my friendships”, “I might lose my job”, “might affect my romantic or sexual relationships”, “I might be excluded from activities”, “they would see or treat me differently”, and “they might then disclose my HIV status to others”.

Prevalence of anticipated stigma was significantly higher among East Asian than non-Asian participants (72.2%[166/230] vs. 63.8%[1377/2159], p = 0.011). This difference remained statistically significant even after adjusting for differences in age, gender, sexual orientation, education, and viral suppression. In the East Asian region, prevalence estimates for anticipated stigma were 58%[32/55] in Taiwan, 72%[36/50] in China, 73%[55/75] in Japan, and 86%[43/50] in South Korea (Fig. 2). A significantly higher percentage of East Asian (68.7%[158/230]) than non-Asian participants (43.3%[935/2159] indicated that someone seeing their HIV pills would cause them much “stress or anxiety” (p < 0.001). Actions taken by some PLHIV to prevent unwanted disclosure included restricting who they shared their HIV status with (percentage who “always” shared their status only 3.9%[9/230] vs. 7.1%[154/2159] among East Asian vs. non-Asian participants respectively), hiding or disguising their HIV pills (77.0%[177/230] vs. 55.9%[1206/2159], respectively), or skipping a dose because of privacy concerns (48.3%[111/230] vs. 27.0%[582/2159], respectively). Of East Asian participants, Taiwanese respondents reported the highest percentage of those who: felt comfortable sharing their HIV status (42%[23/55]), had shared their HIV status with someone besides their primary HIV care provider (89%[49/55]), and felt comfortable discussing privacy-related challenges with their provider (64%[35/55]) (Fig. 2).

However, not all reported stigma was external as a large segment of PLHIV recounted negative perceptions of themselves (i.e., internal stigma). Overall, 50.0%[115/230] of East Asian participants believed HIV would reduce their lifespan (China, 70.0%; Japan, 46.7%; South Korea, 58.0%; Taiwan, 29.1% vs. non-Asian participants, 40.7%) and 43.0%[99/230] no longer even planned for their old age because of HIV (China, 54.0%[27/50]; Japan, 42.7%[32/75]; South Korea, 44.0%[22/50]; Taiwan, 32.7%[18/55] vs. non-Asian participants, 30.7%[663/2159]). Even among those reporting being virally suppressed in the East Asian subgroup, 44.7%[68/152] believed HIV would reduce their lifespan, although this was lower compared to those not reporting viral suppression (60.3%[47/78], p = 0.026). Furthermore, 53.9%[124/230] of East Asian participants felt that their daily HIV medicines reminded them of their HIV, while 42.2%[97/230] said daily ART dosing triggered painful memories. Over a third (36.5%[84/230]) of East Asian participants believed there was room for improving their HIV medicine (Japan, 33.3%[25/75]; South Korea, 34.0%[17/50]; China, 36.0%[18/50]; Taiwan, 43.6%[24/55]).

### Stigma-avoidance Behaviors Among PLHIV

**Fig. 3:**
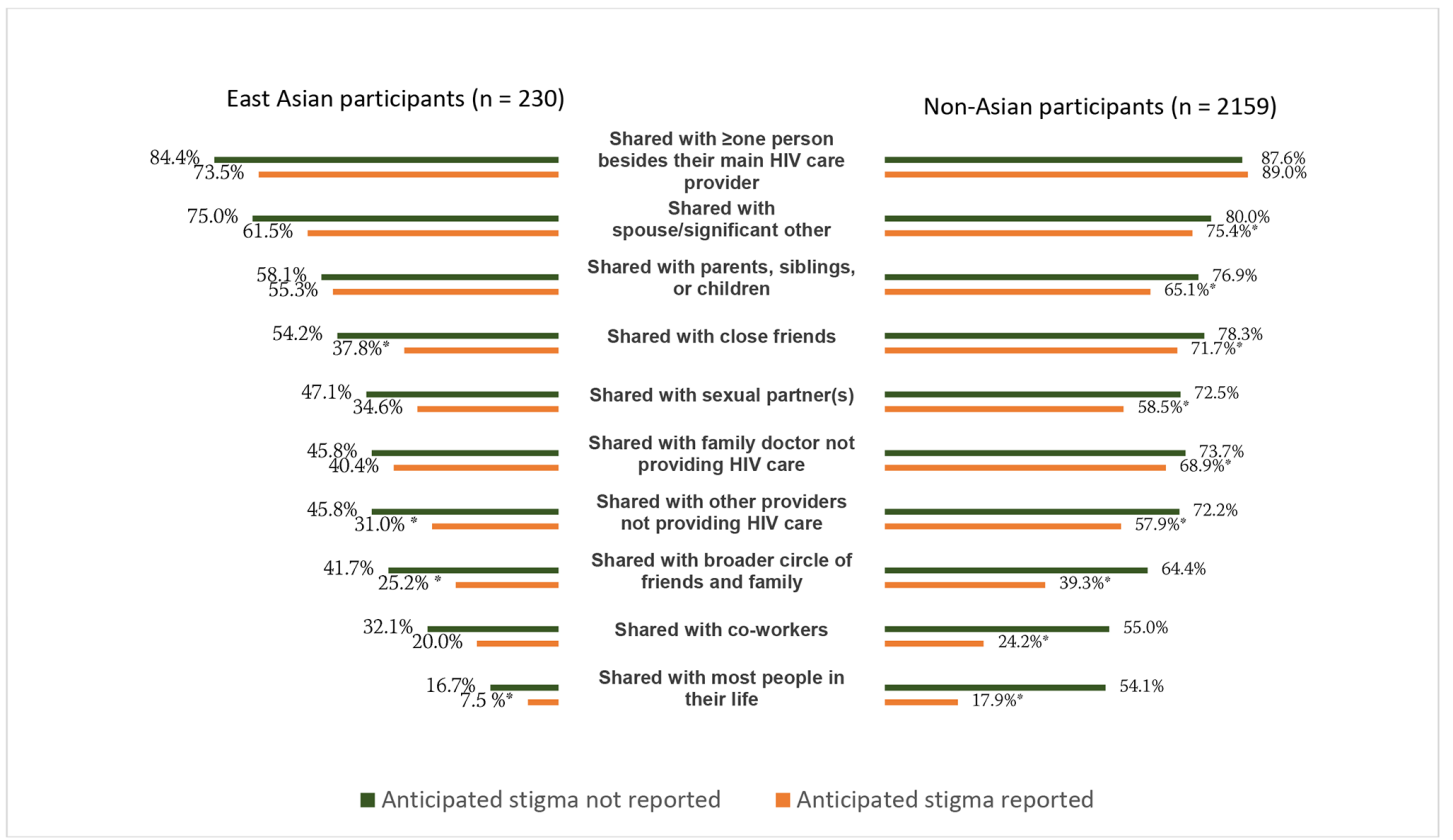
Disclosure of HIV status within different inter-personal relationships among people living with HIV with vs. without a report of anticipated stigma, stratified by East Asian (n = 230) or non-Asian region (n = 2159). (Note: Comparisons are *within* the separate subpopulations of East Asian (reporting anticipated stigma, n = 166 vs. not reporting anticipated stigma, n = 64) and non-Asian participants (reporting anticipated stigma, n = 1377 vs. not reporting anticipated stigma, n = 782), not *between* the East Asian vs. not Asian participants. Participants were asked whether they had shared their HIV status within different relationship types, including with their spouse/significant other; casual sex partners; close friends; close family members such as parents, children, or siblings; extended family/friends; co-workers; healthcare professionals not providing HIV care; and “Most of the people in my life”. Only people in those specified relationships responded (e.g., only those with a spouse/significant other answered the question of whether they had shared with a spouse/significant other); the rest selected “not applicable” and were excluded from those analyses) * P < 0.05.

To prevent being discriminated against, participants commonly withheld their HIV status, even within intimate social circles defined by blood, sex, or marriage (Fig. 3). Compared to those without anticipated stigma within the East Asian subgroup, those with anticipated stigma were less likely to share their HIV status with close friends (37.8%[59/156] vs. 54.2%[32/59], , p = 0.030), healthcare providers not their primary HIV physician or family doctor (31.0%[48/155] vs. 45.8%[27/59], p = 0.043), extended family/friends (25.2%[39/155] vs. 41.7%[25/60], p = 0.018), or with “most people in their life” (7.5%[11/147] vs. 16.7%[10/60], p = 0.047) (Fig. 3).

As shown in Fig. 1, the foremost reason for which East Asian participants refused to share their HIV status was fear of being treated differently (58.7%[135/230]); this did not differ significantly from non-Asian participants (58.6%[1265/2159], p = 0.976). Within the East Asian region, differences existed in refusing to share HIV status for fear of being treated differently (China, 76.0%[38/50]; Japan, 37.3%[28/75]; South Korea, 60.0%[30/50]; Taiwan, 70.9%[39/55], p < 0.001), being the subject of gossip (China, 58.0%[29/50]; Japan, 38.7%[29/75]; South Korea, 48.0%[24/50]; Taiwan, 72.7%[40/55], p = 0.001), being excluded from social activities (China, 56.0%[28/50]; Japan, 36.0%[27/75]; South Korea, 40.0%[20/50]; Taiwan, 61.8%[34/55], p = 0.011), losing their job (China, 28.0%[14/50]; Japan, 33.3%[25/75]; South Korea, 38.0%[19/50]; Taiwan, 63.6%[35/55], p = 0.001), being denied healthcare (China, 18.0%[9/50]; Japan, 16.0%[12/75]; South Korea, 22.0%[11/50]; Taiwan, 52.7%[29/55], p < 0.001), romantic discrimination (China, 26.0%[13/50]; Japan, 30.7%[23/75]; South Korea, 26.0%[13/50]; Taiwan, 56.4%[31/55], p = 0.002), or being the victim of violence (China, 20.0%[10/50]; Japan, 26.7%[20/75]; South Korea, 10.0%[5/50]; Taiwan, 36.4%[20/55], p = 0.013).

Within pooled multivariable analysis of the full dataset, participants in North America had lower odds than those in East Asia to report withholding their HIV status for fear of being gossiped about (adjusted odds ratio [AOR] = 0.68), being denied healthcare services (AOR = 0.66), losing their jobs (AOR = 0.65), being treated differently (AOR = 0.64), or excluded from social activities (AOR = 0.56), (all p < 0.05, Table 2). Participants in Europe also were less worried than those in East Asia of being excluded from activities (AOR = 0.68), physically attacked (AOR = 0.61), denied financial benefits (AOR = 0.56), denied healthcare services (AOR = 0.52), or prosecuted (AOR = 0.52) (all p < 0.05). Participants in Latin America were less likely than those in East Asia to withhold their HIV status for fear of being denied healthcare services (AOR = 0.62) or being prosecuted (AOR = 0.43) (all p < 0.05) but did not differ in other respects. Older adults aged 50 + years were less likely than younger ones to withhold their HIV status out of fear of losing their jobs (AOR = 0.80), being gossiped about (AOR = 0.79), or suffering romantic rejection (AOR = 0.71) (all p < 0.05). When compared to people identifying as MSW, those identifying as MSM had higher odds of withholding their HIV status for fear of being gossiped about (AOR = 1.34), losing their jobs (AOR = 1.41), being treated differently (AOR = 1.55), or suffering romantic rejection (AOR = 1.78); they were however less likely to worry about criminal prosecution because of their HIV status (AOR = 0.60) (all p < 0.05). Women were more worried than MSW that disclosing their HIV status could lead to losing their jobs (AOR = 1.51), like MSM however, they (women) were less worried about criminal prosecution when compared to MSW (AOR = 0.56) (all p < 0.05). Compared to those living in non-metropolitan areas, those living in metropolitan areas had higher odds of not sharing their HIV status for fear they might be gossiped about (AOR = 1.31) or treated differently (AOR = 1.46) (both p < 0.05). When compared to those neither virally suppressed nor aware of U = U, those reporting they were both virally suppressed and had been told of U = U by their healthcare provider reported lower odds of withholding their HIV status for fear of losing their jobs (AOR = 0.70), being excluded from social activities (AOR = 0.65), or being physically attacked (AOR = 0.63); conversely, they were more likely to have ever withheld their HIV status to avoid romantic discrimination (AOR = 1.45) (all p < 0.05).

**Table 2:** Factors associated with different reasons reported for nondisclosure of HIV status among people living with HIV in all study locations (N = 2389)

	Worried people would see or treat me differently	Worried people might then disclose my HIV status to others	Worried that I might be excluded from activities	Worried about being denied access to health care services	Worried about being denied access to financial benefits	Worried it might affect my friendships	Worried I might lose my job	Worried it might affect my romantic or sexual relationships	Worried about my physical safety/ potential violence	Worried about criminal prosecution
**Age, y**										
< 50 y (reference group)										
50+	0.85 (0.70–1.05)	0.79 (0.65–0.97) *	0.85 (0.69–1.05)	1.00 (0.77–1.30)	0.92 (0.71–1.21)	0.97 (0.80–1.18)	0.80 (0.65–0.99) *	0.71 (0.58–0.88) *	0.87 (0.67–1.12)	0.70 (0.49–1.01)
**Gender/sexual orientation**										
Men who have sex with women (reference group)										
Men who have sex with men	1.55 (1.21–1.98) *	1.34 (1.05–1.71) *	1.10 (0.86–1.41)	0.79 (0.58–1.08)	1.03 (0.75–1.40)	1.26 (0.99–1.61)	1.41 (1.08–1.83) *	1.78 (1.38–2.31) *	1.17 (0.86–1.58)	0.60 (0.42–0.87) *
Women	1.18 (0.92–1.51)	0.99 (0.77–1.27)	1.08 (0.84–1.39)	1.12 (0.83–1.52)	0.92 (0.67–1.26)	1.14 (0.90–1.46)	1.51 (1.17–1.96) *	0.89 (0.68–1.17)	1.04 (0.77–1.41)	0.56 (0.39–0.82) *
Undisclosed or unknown	1.21 (0.85–1.72)	1.01 (0.71–1.43)	0.80 (0.56–1.15)	0.66 (0.41–1.08)	0.95 (0.60–1.51)	0.93 (0.66–1.31)	0.91 (0.62–1.34)	1.22 (0.85–1.75)	1.31 (0.86–1.98)	0.57 (0.31–1.04)
**Region**										
East Asia										
Northern America	0.64 (0.46–0.88) *	0.68 (0.49–0.93) *	0.56 (0.41–0.78) *	0.66 (0.46–0.97) *	0.84 (0.58–1.22)	0.73 (0.53–1.01)	0.65 (0.46–0.90) *	0.71 (0.50-1.00)	0.85 (0.58–1.24)	0.93 (0.60–1.45)
Europe	0.99 (0.73–1.34)	0.87 (0.64–1.17)	0.68 (0.51–0.92) *	0.52 (0.37–0.75) *	0.56 (0.39–0.80) *	0.80 (0.60–1.08)	0.80 (0.59–1.09)	0.93 (0.68–1.28)	0.61 (0.43–0.88) *	0.52 (0.34–0.81) *
Latin America	1.18 (0.80–1.74)	1.46 (1.00-2.15)	0.85 (0.58–1.24)	0.62 (0.39–0.98) *	0.65 (0.41–1.04)	0.76 (0.52–1.10)	1.16 (0.79–1.71)	1.46 (0.99–2.15)	1.38 (0.90–2.11)	0.43 (0.23–0.82) *
Other	1.06 (0.76–1.48)	1.10 (0.79–1.52)	0.60 (0.43–0.84) *	0.79 (0.54–1.15)	0.45 (0.30–0.69) *	0.86 (0.62–1.20)	0.63 (0.45–0.89) *	1.30 (0.93–1.83)	0.81 (0.54–1.19)	0.41 (0.24–0.71) *
**Year of HIV diagnosis**										
2017 to 2019 (reference group)										
2010 to 2016	0.97 (0.78–1.22)	1.12 (0.90–1.40)	0.86 (0.69–1.08)	0.78 (0.60–1.03)	0.80 (0.61–1.06)	0.97 (0.78–1.21)	0.87 (0.70–1.10)	1.05 (0.83–1.33)	1.00 (0.77–1.31)	0.93 (0.66–1.31)
Pre-2010	1.02 (0.79–1.30)	1.07 (0.83–1.36)	0.93 (0.72–1.19)	0.85 (0.62–1.16)	0.89 (0.65–1.22)	0.96 (0.75–1.22)	0.95 (0.73–1.23)	1.27 (0.98–1.64)	1.10 (0.81–1.49)	1.10 (0.73–1.65)
**Domicile**										
Non-metropolitan (reference group)										
Metropolitan	1.46 (1.23–1.74) *	1.31 (1.10–1.56) *	1.03 (0.86–1.23)	1.12 (0.90–1.41)	0.86 (0.68–1.07)	1.16 (0.98–1.38)	0.97 (0.81–1.17)	1.13 (0.94–1.35)	0.92 (0.74–1.15)	0.88 (0.66–1.18)
**Education**										
≤ High school										
> High school	0.82 (0.67–1.01)	0.90 (0.73–1.10)	0.89 (0.72–1.09)	1.21 (0.92–1.59)	1.11 (0.85–1.46)	0.88 (0.72–1.08)	1.09 (0.88–1.35)	0.99 (0.80–1.22)	0.84 (0.66–1.08)	1.25 (0.87–1.78)
Prefer not to answer	2.07 (1.25–3.44) *	2.56 (1.56–4.19) *	2.32 (1.48–3.64) *	1.25 (0.69–2.27)	1.27 (0.69–2.33)	1.34 (0.86–2.09)	0.97 (0.59–1.62)	1.63 (1.04–2.56)	1.05 (0.59–1.87)	0.91 (0.39–2.15)
**U = U awareness and viral status**										
Neither aware of U = U nor virally suppressed (reference group)										
Aware of U = U but not virally suppressed	1.00 (0.72–1.39)	0.75 (0.53–1.04)	1.00 (0.72–1.39)	1.11 (0.74–1.68)	1.56 (1.03–2.36) *	0.92 (0.66–1.27)	1.30 (0.93–1.82)	1.09 (0.75–1.59)	1.30 (0.89–1.89)	1.58 (0.97–2.58)
Unaware of U = U but virally suppressed	0.96 (0.70–1.33)	1.10 (0.80–1.51)	0.72 (0.52-1.00)	1.35 (0.91–1.99)	1.35 (0.89–2.05)	0.78 (0.57–1.07)	0.93 (0.67–1.29)	1.28 (0.90–1.82)	0.93 (0.64–1.36)	1.15 (0.69–1.91)
Both aware of U = U and virally suppressed	1.03 (0.76–1.39)	1.09 (0.81–1.47)	0.65 (0.48–0.88) *	0.86 (0.59–1.26)	1.04 (0.70–1.54)	0.76 (0.57–1.03)	0.70 (0.51–0.96) *	1.45 (1.05–2.02) *	0.63 (0.44–0.90) *	1.03 (0.64–1.66)

Note: Adjusted odds ratios were adjusted for all factors listed in the table. U = U is Undetectable Equals Untransmittable. * p < 0.05

Of East Asian participants, those anticipating stigma within healthcare facilities (n = 61) were significantly less likely to communicate freely with their healthcare providers on salient treatment issues, compared to those not anticipating stigma in healthcare settings (n = 169) (Table 3). For example, the former felt less comfortable discussing with their healthcare provider how to prevent HIV transmission to others (37.7%[23/61] vs. 55.0%[93/169], p = 0.02) as well as how to manage HIV-related illnesses (47.5%[29/61] vs. 63.3%[107/169], p = 0.032). They were also more likely to report certain communication barriers, such as fear of being labelled a “difficult patient” (49.2%[30/61] vs. 33.7%[57/169], p = 0.033) and the perception that there was never enough time or opportunity during their visit (36.1%[22/61] vs. 18.9%[32/169], p = 0.007).

**Table 3:** Percentage of people living with HIV in the East Asian region who reported various indicators of communication with their healthcare providers, stratified by whether they reported anticipated stigma within healthcare settings (N = 230)

Indicators	Overall	Among those with no report of ever refusing to share their HIV status for fear of losing their healthcare benefits	Among those with a report of ever refusing to share their HIV status for fear of losing their healthcare benefits	Chi square statistic	P-value
**Perceived comfort discussing salient treatment concerns**	(N = 230)	(N = 169)	(N = 61)		
The impact HIV is having on my life generally	50.0	52.7	42.6	1.8072	0.179
The safety of others/preventing transmission	50.4	55.0	37.7	5.3816	0.020 *
My emotional well-being	50.0	51.5	45.9	0.5578	0.455
Privacy and not disclosing my HIV status	47.4	48.5	44.3	0.326	0.568
Having children	37.4	39.1	32.8	0.7518	0.386
Illnesses caused by HIV	59.1	63.3	47.5	4.6141	0.032 *
Side effects of my HIV medicine	63.5	66.3	55.7	2.1456	0.143
How my HIV medicine affects other medicines/drugs/pills I take	55.7	59.2	45.9	3.198	0.074
Concerns about long-term side effects of my HIV medicine	66.1	67.5	62.3	0.5326	0.466
Skipping/missing medicine or forgetting to take my pill(s) each day	50.4	53.3	42.6	2.0266	0.155
**Perceived barriers to quality communication with providers**					
I feel my main HIV care provider knows best	20.4	21.3	18.0	0.2946	0.587
I don’t feel confident enough	31.3	29.0	37.7	1.5815	0.209
There never seems to be enough time or the opportunity	23.5	18.9	36.1	7.3213	0.007 *
I’m not sure how to bring it up	33.5	33.1	34.4	0.0335	0.855
I don’t want to take up more of their time	22.6	21.3	26.2	0.622	0.430
I don’t feel it is important enough to bother them	21.7	20.1	26.2	0.9839	0.321
I don’t want to come across as a ‘difficult’ patient	37.8	33.7	49.2	4.5508	0.033 *
I don’t believe they can do much about my concerns	33.0	32.0	36.1	0.3427	0.558
I don’t think my main HIV care provider’s priorities are the same as mine	27.0	26.6	27.9	0.0351	0.851
**Reported extent of engagement by providers**					
I am given enough information to be involved in making treatment choices	62.2	62.1	62.3	0.0005	0.982
I feel I understand enough about my HIV treatment	62.2	62.7	60.7	0.0814	0.775
My provider seeks my views about treatment before prescribing an HIV medicine	63.0	65.7	55.7	1.9018	0.168
My provider asks me if I have any concerns about the HIV medicine I am currently taking	65.2	65.7	63.9	0.0602	0.806
My provider tells me about new HIV treatment options that become available	58.7	61.5	50.8	2.1241	0.145
My provider asks me frequently about any side effects I might be experiencing	58.3	58.6	57.4	0.0267	0.870
My provider has told me about “Undetectable = Untransmittable” (U = U)	51.3	50.9	52.5	0.0443	0.833
My provider meets my needs and considers things that are important to me	57.4	59.8	50.8	1.4661	0.226
**Other indicators of patient-provider engagement**					
I would like to be more involved when it comes to decisions about my HIV treatment	68.7	67.5	72.1	0.4556	0.500
Have ever told their provider of a new treatment they wanted different from what they were on	51.7	56.2	39.3	5.1079	0.024 *
Have shared their HIV status with their family doctor not providing HIV care	41.9	43.9	36.4	0.9398	0.332
Have shared their HIV status with other providers other than their family doctor or main HIV care provider	35.0	35.4	33.9	0.0417	0.838

* p < 0.05 or statistically significant differences

* P < 0.05

### The Relationship Between Reported Stigma and Treatment Preferences

**Fig. 4:**
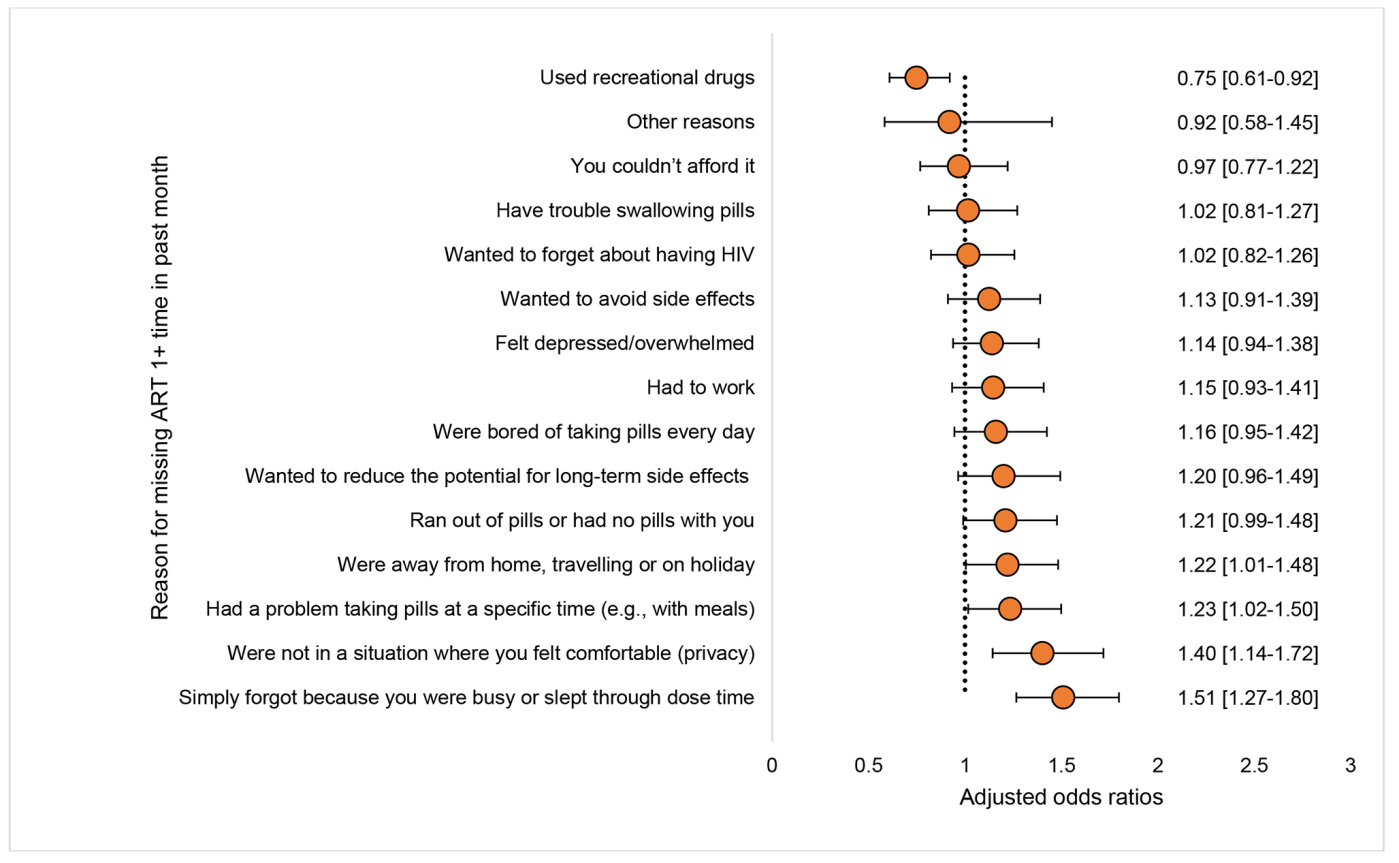
Adjusted odds of missing HIV medicines for specific reasons when comparing people living with HIV with vs. without a report of anticipated stigma using pooled data from all participating locations (N = 2389). (Note: Analysis adjusted for geographic region, HIV duration, and gender)

**Fig. 5:**
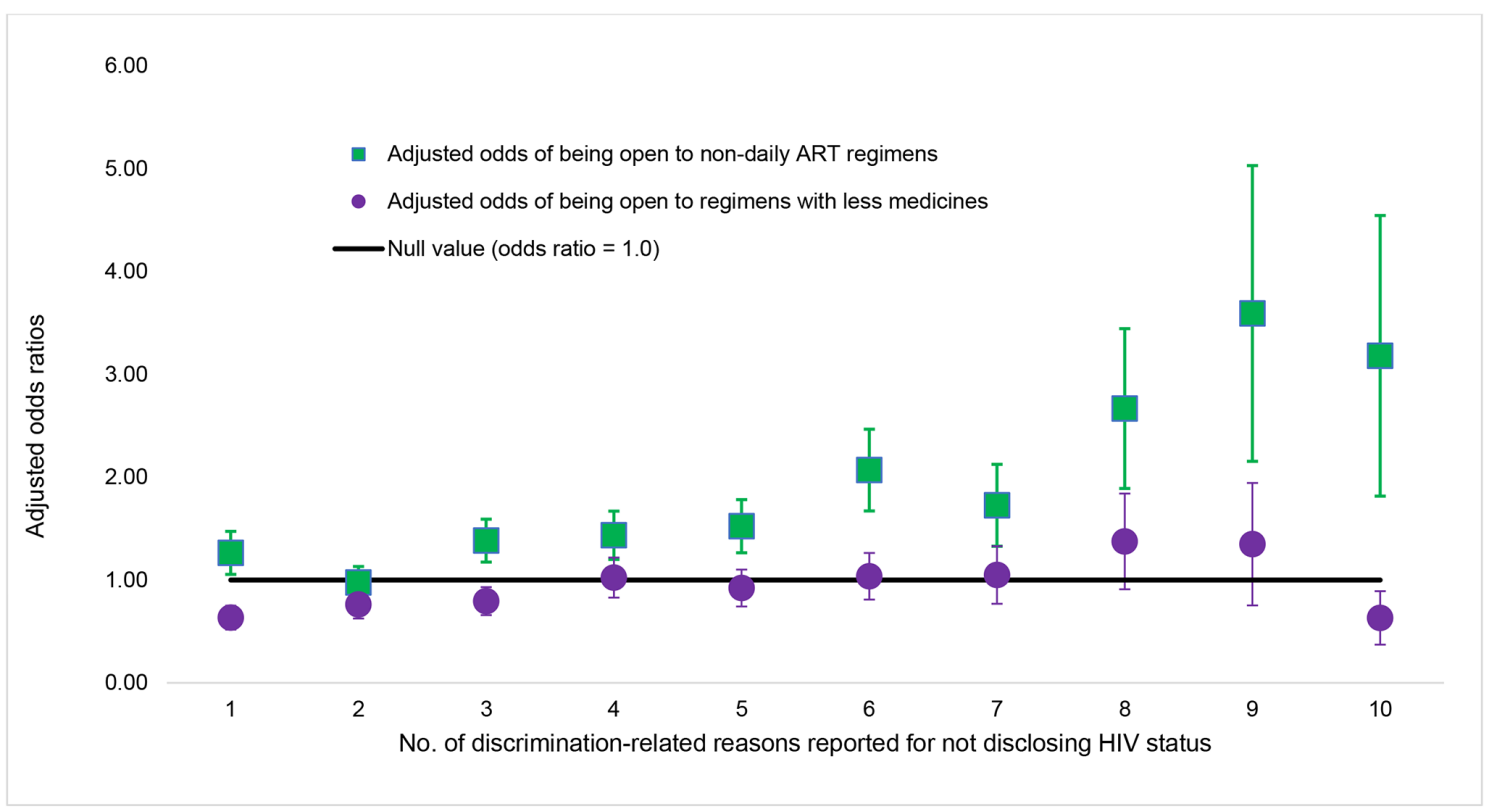
Adjusted odds ratios with corresponding 95% confidence intervals showing how openness to the specified novel treatments changed with increasing number of stigma-related stressors among people living with HIV in all participating locations (N = 2389). (Note: As a proxy for the number of stigma stressors, we tallied the different contexts in which participants refused to share their HIV status to avoid discrimination (range: 0 to 10). Adjusted odds ratios were calculated in a binary logistic regression model and those with a tally of 0 are the reference group (i.e., individuals who freely shared their HIV status under all the various contexts assessed). Analyses adjusted for geographic region, duration of HIV, and gender)

Figure 4 shows the distinctive reasons for poor adherence reported among those with vs. without anticipated stigma in the full dataset from all 25 locations, while Fig. 5 shows which treatment modalities were prioritized the most by those with anticipated stigma. When comparing reasons for missing ART at least once in the past month between those with vs. without anticipated stigma in the full dataset, the reasons that emerged as significantly predicting poorer adherence among those with anticipated stigma were being on travel or on holidays (AOR = 1.22), problems with dosing at a specific time (AOR = 1.23), privacy concerns (AOR = 1.40), and being busy or sleeping through dose time (AOR = 1.51) (all p < 0.05).

PLHIV overall were receptive to novel treatments with 57.8%[133/230] of East Asian participants (54.3%[1173/2159] non-Asian, p = 0.311) indicating openness to non-daily ART, and 65.2%[150/230] of all East Asian participants (73.0%[1576/2159] non-Asian, p = 0.012) indicating openness to ART with fewer medicines. Anticipated stigma was strongly associated with receptivity to non-daily regimens. As shown in Fig. 4, with increasing number of discrimination-related reasons reported for not sharing HIV status, there was a marked increase in the odds of being open to non-daily ART, compared to those not reporting a single discrimination-related reason for nondisclosure. For example, compared to the latter, the odds of preferring a non-daily regimen were elevated among those reporting the following number of discrimination-related reasons for nondisclosure: three reasons (AOR = 1.38), four (AOR = 1.44), five (AOR = 1.52), six (AOR = 2.07), seven (AOR = 1.73), eight (AOR = 2.67), nine (AOR = 3.59), or ten reasons (AOR = 3.18) (all p < 0.05). Despite these unique unmet needs, those with anticipated stigma were less likely to communicate their preferred treatments with their providers. In the East Asian region, for example, only 39.3%[24/61] of those anticipating stigma in healthcare settings had ever told their healthcare provider of a new medicine they wanted, vs. 56.2%[95/169] of those without anticipated stigma in healthcare settings (p = 0.024).

## Discussion

Prevalence of anticipated stigma was significantly higher among Asian than non-Asian participants with the difference persisting even after adjusting for several sociodemographic and clinical correlates, suggesting the role of other underlying contextual factors (e.g., cultural norms). Many of the underlying reasons in our study for the unwillingness of PLHIV to share their HIV status appear well founded in lived experiences as previous research in the Asian region reported some of these very same negative experiences happening to PLHIV in the past 12 months. For example, the 2011 UNAIDS report indicated that 12% of surveyed PLHIV reported being “denied health services (including dental care) because of HIV status in past 12 months”; [27] in our study, 18% of Chinese participants reported being hesitant of sharing their HIV status with others for fear of being denied healthcare services. Our results showed that anticipated stigma was not only associated with internalized stigma (e.g., expressing sentiments they would die prematurely from HIV), but also with treatment avoidance behaviors to avoid inadvertent disclosures. This underscores the need to reduce HIV-related stigma and to address the unmet needs of PLHIV that contribute to stigma, including medical, economic, and psychosocial challenges. [23].

Opportunities exist within the health system to address both institutional and internalized stigma. U = U education could be incorporated into continuing medical education for healthcare providers as a way of reducing institutional stigma within healthcare facilities. This way, the U = U message could benefit PLHIV both directly (empowering them and reducing internalized stigma), as well as indirectly (reducing external stigma from healthcare settings). In our study, participants who were both virally suppressed and had been counselled by their providers about U = U were more empowered to share their HIV status in various situations. Incorporating U = U counselling into clinical practice guidelines and protocols can increase its adoption and implementation within clinical settings. Healthcare providers can also help their patients mitigate internalized stigma by carefully considering newer treatment options that might address PLHIV’s confidentiality concerns. Our finding of a strong relationship between openness to long-acting treatment regimens and number of stigma incidents is important and may have implications on disseminating treatment and person-centered clinical practice.

Participants in East Asia reported significantly higher stigma than the rest of the sampled population mostly from Europe and North America. This finding could be attributable to differential injunctive social norms or perceived acceptability surrounding HIV diagnosis; in areas with high HIV deaths, HIV could be perceived as a death sentence. For example, while the number of PLHIV in Southeast Asia during 2019 was only 1.59 times higher than in North America (2.91 vs. 1.83 million respectively), the number of HIV deaths in that same year was over 10 times higher in Southeast Asia (76,748 deaths) than North America (7,312 deaths). [25] HIV stigma may also be driven by an unfavorable social climate towards same-sex relationships—a grave concern given that MSM are a key demographic among those living with HIV in the Asian region [41]. Many PLHIV may therefore be exposed to co-occurring and amplified stigmas where the identities of their HIV status and their sexual orientation converge. The overwhelming majority of countries and territories that have enacted national laws allowing gays and lesbians to marry are in Europe and the Americas; many in the Asian region are not covered by such laws. [42] Not to be overlooked also are HIV criminalization laws which are widespread in the Asian region [43] as well as higher co-prevalence of other stigmatizing conditions such as tuberculosis among PLHIV relative to North America or Europe. [25] To accelerate progress in eradicating AIDS as a public health threat by 2030 [44,45], concerted efforts must be made towards achieving not only clinical or patient-level targets (e.g., 95-95-95), but also ensuring that significant gains are made in the policy space to roll-back discriminatory laws and policies that directly or indirectly engender, perpetuate, or reinforce HIV stigma.

Addressing stigma within healthcare settings is particularly important as this has a direct and measurable impact on patient-provider communication. Among participants from the Asian region in our study, we observed that those who anticipated stigma within healthcare settings were less likely to discuss certain salient issues with their HIV care providers, such as how to prevent HIV transmission to others and how to manage HIV-related illnesses. Comprehensive efforts to address stigma within healthcare settings should include systematic interventions to flag and address seemingly innocent, but misguided values, behaviors, attitudes, or speech that could be perceived as discriminatory among PLHIV.[9,46-49] Improving the quality of communication between patients and their HIV healthcare providers and between different providers co-managing the patient may accelerate progress towards the goal of improving health-related quality of life among PLHIV.

This study’s novelty lies in exploring the association between extent of stigma and preference for newer HIV medications that have potential to reduce confidentiality concerns among PLHIV (e.g., long-acting injectable regimens for HIV treatment which are less frequent and more discreet). An additional strength of the study is its use of a standardized protocol in 25 countries. To the best of our knowledge, this is one of the largest studies of PLHIV to assess patient-centered outcomes, including aspirations and attitudes toward treatment. Nonetheless, some limitations exist. First, participants were selected non-probabilistically, which may limit the generalizability of the study findings to both the individual countries and the East Asian region. [50] Second, these data are cross-sectional, so only associations can be drawn. Third, all measures were self-reported and may be subject to various social and cognitive biases and result in misclassification. Fourth, our study did not evaluate stigma in all its forms; similarly, data were not collected on some key demographic groups, including bisexual men. Finally, the measurement of stigma was done using surrogate markers because of the absence of validated measures within the analyzed dataset. While this limits direct comparability with other studies that have used such validated markers, it is reasonable to assume that stigma was adequately captured by these surrogate markers given that the indicators are similar to those in psychometrically evaluated scales. It has been suggested that generic stigma measures can indeed provide an accurate assessment of how people experience stigma. [51] Despite these limitations, this study provides important data for better understanding stigma among PLHIV from a regional and global perspective.

## Conclusion

Stigma in different forms was common among Asian PLHIV and was associated with poor patient-provider communication. In addition, anticipated stigma was associated with a greater preference for non-daily regimens. A holistic consideration of psychosocial and emotional outcomes, beyond virologic control, may improve health-related quality of life among PLHIV. Furthermore, increasing flexibility of ART delivery and improved treatment choices may improve PLHIV’s adherence to treatment while addressing unmet needs associated with HIV confidentiality and stigma. Addressing HIV stigma in all its forms – experienced, anticipated, or internalized, is critical to meeting the 2030 goal of eradicating HIV as a public health threat.
